# Hand grip strength should be normalized by weight not height for eliminating the influence of individual differences: Findings from a cross-sectional study of 1,511 healthy undergraduates

**DOI:** 10.3389/fnut.2022.1063939

**Published:** 2023-01-18

**Authors:** Taojin Xu, Xu Li, Dingfang Wang, Yi Zhang, Qinghua Zhang, Jianyin Yan, Junhao Jiang, Wenbin Liu, Jing Chen

**Affiliations:** ^1^School of Advanced Manufacturing Engineering, Chongqing University of Posts and Telecommunications, Chongqing, China; ^2^Key Laboratory of Big Data Intelligent Computing, Chongqing University of Posts and Telecommunications, Chongqing, China; ^3^College of Physical Education, Chongqing University of Posts and Telecommunications, Chongqing, China; ^4^The Fifth People’s Hospital of Chongqing, Chongqing, China

**Keywords:** hand grip strength, adolescent, forearm circumference, correlation, muscular function

## Abstract

**Background:**

Hand grip strength (HGS) is a fast, useful, and inexpensive outcome predictor of nutritional status and muscular function assessment. Numerous demographic and anthropometric factors were reported to be associated with HGS, while which one or several factors produce greater variations in HGS has not been discussed in detail. This is important for answering how should HGS be normalized for eliminating the influence of individual differences in clinical practice.

**Aims:**

To compare the contribution of age, sex, height, weight, and forearm circumference (FCF) to variations in HGS based on a large-scale sample.

**Methods:**

We enrolled 1,511 healthy undergraduate students aged 18–23 years. Age, weight, height, and sex were obtained. HGS was measured using a digital hand dynamometer, and FCF was measured at the point of greatest circumference using a soft ruler in both hands. Pearson’s or Spearman’s correlation coefficients were calculated with data of women and men separated and mixed for comparison. Partial correlation analysis and multivariate linear regression were used to compare the effect of variables on HGS.

**Results:**

Analysis results confirmed the correlates of higher HGS include higher height, heavier weight, being men and dominant hand, and larger FCF. The correlation between HGS and FCF was the highest, and the bivariate correlation coefficient between weight and HGS was largerata of women and men were mixed, than that between height and HGS. When data of women and men were mixed, there were moderate correlations between HGS and height and weight (*r* = 0.633∼0.682). However, when data were separated, there were weak correlations (*r* = 0.246∼0.391). Notably, partial correlation analysis revealed no significant correlation between height and HGS after eliminating the weight effect, while the correlation between weight and HGS was still significant after eliminating the height effect. Multivariate linear regression analyses revealed sex was the most significant contributor to the variation in HGS (Beta = –0.541 and –0.527), followed by weight (Beta = 0.243 and 0.261) and height (Beta = 0.102 and 0.103).

**Conclusion:**

HGS and FCF reference values of healthy college students were provided. Weight was more correlate with hand grip strength, at least among the healthy undergraduates.

**Clinical trial registration:**

http://www.chictr.org.cn/showproj.aspx?proj=165914, identifier ChiCTR2200058586.

## Highlights

– HGS and FCF reference values of healthy college students were provided.
– Mixing data of women and men confounded previous correlation analyses.
– Weight is more correlate with HGS than height among healthy college students.

## 1. Introduction

Hand grip strength (HGS) is commonly used for nutritional status and muscular function assessment (1–3). It is a key component of Frailty and Sarcopenia syndromes (4–7). Previous studies have reported that factors associated with HGS include demographic factors, anthropometric variables, level of physical activity, cognitive status (3, 8–10), and so on. However, many of these factors are interrelated, such as the positive correlation between height and weight. Ergonomic studies also observed that arm circumference is significantly related to height, weight, and body mass index (BMI) (3, 11–14). Currently, the contribution of age, sex, height, weight, and forearm circumference (FCF) to variations in HGS has not been discussed in detail. This is important to answer whether HGS should be stratified by height, weight, or BMI, to explain the regional or individual differences (15). Some studies chose to normalize HGS by body mass (16, 17), while others preferred to adjust HGS by height (18) or BMI (19).

Previous studies have shown contradictory results on the correlation coefficient between HGS and height and weight. Some studies reported that the correlation coefficient between HGS and height is larger than that between HGS and weight (3, 20–28). In other studies, the correlation coefficient between HGS and height is, on the contrary, smaller than that between HGS and weight (12, 29–31). Other studies reported that the correlation coefficient is similar (10, 32, 33) or smaller in women while larger in men (34). The contradiction can be partly explained by the differences in the study population and the data analysis method. Some studies focused on children and adolescents (22–24, 26, 30, 32), mature young people (12, 29, 33), young adult and middle-aged subjects (10) and older adults (20), while others focused on people across all the life course (3, 21, 25–28, 31). As for the data analysis method, most studies combined data from women and men to calculate the correlation coefficient between HGS and height and weight (3, 10, 12, 20, 28, 32, 33). However, age and sex are the strongest influencing factors on HGS (1, 8). Therefore, previous studies may not reveal the actual correlation between HGS and height and weight.

In addition, published reference data of HGS and FCF are primarily derived from Caucasian populations in high-income countries and partly from East China (30, 35), with no data derived from Southwest China. Therefore, this study aimed to provide HGS and FCF reference values of healthy undergraduate students, and to analyze the contribution of age, sex, height, weight, and FCF to variations in HGS based on a large-scale cohort of healthy undergraduate students.

## 2. Materials and methods

### 2.1. Study population

This study was carried out in two universities in Southwest China. The first wave was carried out on the Chongqing University of Posts and Telecommunications campus from April to June 2022, and the second wave was on the Chongqing College of International Business and Economics campus from October to November 2022. Participants were healthy undergraduate students aged 18∼23 years and lived in both rural and urban areas of China but were mainly from Southwest China. Participants were recruited in their physical education courses on the playground or in the fitness room. In each measurement, there were a large number of classes having physical education courses. The researchers randomly selected part of those classes to complete the measurements. The exclusion criteria were pain or restriction of movement of a hand or arm, neuromuscular disease, generalized bone disease, aneuploidy, any condition that severely interfered with normal growth or required hormonal supplementation, and participants who were unwilling to participate in this experiment.

All the participants gave their written informed consent, and the study was conducted in accordance with the regulations. This study was approved by the Institutional Review Board of the Chongqing University of Posts and Telecommunications and was registered in the Chinese Clinical Trial Registry (No. ChiCTR2200058586). Registration information can be accessed here: http://www.chictr.org.cn/showproj.aspx?proj=165914. The ‘Clinical study on resistance training of elastic band’ measured age, sex, height, weight, HGS and FCF of left and right hands. This paper reported the results of the pilot analysis of these data.

### 2.2. Collection of basic demographic information

Each measurement session started with a short lecture by the physical education teachers at the College of Physical Education to introduce the researchers to the students, ensuring that all students were willing to cooperate with the researchers’ instruction. One researcher explained the purpose of the study and the experimental procedure. Another researcher helped to distribute pens, informed consent forms and experimental record forms and helped to record experimental data. First, the participants were instructed to read carefully and sign the informed consent form, then fill in their names, heights, weights, ages, dominant hands (DHs) and sex in the record form. The data on height and weight came from their National Student Fitness Test, in which height and weight were measured by the physical education teachers using an ultrasonic height and weight measuring device made in China.

### 2.3. Grip strength and forearm circumference measurements

The Standardized Procedure and Script for Grip Strength and Forearm Circumference Measurement (Version 1.0) used in this study is in the Supplementary material Appendix A. The procedure was approved by the Institutional Review Board of the Chongqing University of Posts and Telecommunications. Our HGS measurement procedure was adapted from the standardized procedure and script for muscle strength testing by Warden (36).

The HGS in kilograms was measured using a digital hand dynamometer (EH101, CAMRY, Guangdong, China) with a resolution of 0.1 kg. The participant stood upright with the arms unsupported parallel to the body and the dynamometer pointing to the ground. In each measurement, the Knob was adjusted to the appropriate position according to the size of the participant’s hand. Participants were instructed to grip the dynamometer as hard as possible for approximately 3 s with both bands. The measurement was repeated for up to three trails if the first two trails did not provide reasonable data (bigger than 10 kg for a healthy woman undergraduate and bigger than 15 kg for a healthy man undergraduate) and allowed 30 s to pass before repeating the measurement. The experimenter then recorded the highest measurement.

The FCF was measured in centimeters using a soft ruler while the participant stood upright with the upper limbs relaxed. The experimenter helped draw out the soft ruler and insert the pluggable pin into the hole to form a ring around the largest circumference of the participant’s forearm and pressed the rebound button to attach the soft ruler to the forearm closely. Both hands were measured, and the experimenter helped to read the scale on the soft ruler and to record it on the record form. All the equipment used in the data collection were calibrated and validated.

### 2.4. Statistical analysis

Descriptive statistics were used to summarize the participants’ main characteristics and experimental performances, including mean, median, interquartile range, frequencies, percentages, and percentile. Statistical analyses were performed separately for women and men, DH and non-dominant hand (NDH), to allow for sex and hand differences, or the overall sample, for comparation. All analyses were performed in SPSS (IBM, Armonk, NY, USA, Version 23), and the results were accepted to be significant when the two-tailed *p*-value was at or less than 0.005 (37).

1. Continuous variables between two groups were compared with the student’s t test or Mann-Whitney U test, if approximate, to determine the significance of differences.
2. The Pearson or Spearman correlation analysis was performed to obtain the univariate correlation coefficients between all variables.
3. The partial correlation analysis was performed to compare the influence of height, weight and FCF on HGS.
4. The multiple linear regression analysis was performed to explore the influence of height, weight, age, and sex on HGS and to establish the formula correlation models. In the regression analysis, HGS was used as the dependent variable, and height, weight, age, and sex were used as the independent variables.
5. Different levels of correlation coefficient (*r*) were defined as: *r* < 0.1 indicates that the correlation is negligible; 0.1 ≤ *r* < 0.4 indicates weak correlation; 0.4 ≤ *r* < 0.7 indicates moderate correlation; 0.7 ≤ *r* < 0.9 indicates strong correlation; *r* > = 0.9 indicates very strong correlation.

## 3. Results

### 3.1. Participants’ basic demographics and experimental performances

A total of 1,527 students participated in this study. Sixteen experimental records were excluded in the final data entry and processing stage due to incomplete information. The final study population comprised 1,511 students (554 women and 957 men) aged 18∼23 years. The proportion of men was high because of the larger proportion of male students in the Chongqing University of Posts and Telecommunications. The basic demographics of participants stratified by sex were summarized in Table 1. According to the interquartile range, there was a slight skewness in the height and weight distribution. Most participants were aged 19∼21 years (accounting for about 80% of the total participants), and the mean age was 20.53 years for women and 19.98 years for men. When compared with women, men were significantly taller (*p* < 0.001) and heavier (*p* < 0.001). Notably, the proportion of overweight (BMI > = 25) in men was higher than that in women and the proportion of underweight (BMI < 18.5) in women was higher than that in men.

**TABLE 1:** Demographic profile of all participants stratified by sex and age (*n* = 1,511).

Parameter	Women	Men
**No. of participants (%)**		
All	554 (36.66)	957 (63.34)
DH is right hand	536 (97.75)	920 (96.13)
Aged 18	19 (3.43)	90 (9.40)
Aged 19	75 (13.54)	243 (25.39)
Aged 20	187 (33.75)	316 (33.02)
Aged 21	162 (29.24)	227 (23.72)
Aged 22	89 (16.07)	67 (7.01)
Aged 23	22 (3.97)	14 (1.46)
BMI < 18.5	150 (27.07)	102 (10.66)
18.5 ≤ BMI < 25	386 (69.68)	719 (75.13)
25 < BMI	18 (3.25)	136 (14.21)
**Height, cm**		
Mean	161.53	174.77
Median (IQR)	161 (158∼165)	175 (171∼178)
Standard deviation	5.19	5.49
Maximum	177	193
Minimum	145	160
*P*-value (*Z*)*	0.000 (–29.82)	
**Weight, kg**		
Mean	52.12	67.18
Median (IQR)	51 (47.475∼56)	65 (60∼73)
Standard deviation	6.70	10.69
Maximum	80	131
Minimum	39	40
*P*-value (*Z*)*	0.000 (–25.94)	

The mean age of women was 20.53 years, and the median (IQR) was 20 (19∼21) years. The mean age of men was 19.98 years, and the median (IQR) was 20 (19∼21) years. Definition of abbreviation: DH, dominant hand; BMI, body mass index; IQR, interquartile range.

*Men vs Women.

Table 2 presents the experimental performances of participants in the HGS and FCF measurements stratified by sex and side of the hand. We compared results between different sex and hand groups. As expected, men were significantly stronger than women with the DH (*p* < 0.001) and NDH (*p* < 0.001), and their FCF was significantly higher accordingly (*p* < 0.001). Both HGS and FCF of DH were significantly higher than NDH except for the FCF of women participants (*p* = 0.014).

**TABLE 2:** Experimental performances in the hand grip strength and forearm circumference measurements stratified by sex and hand.

	Women		Men		*P*-value (*Z*)**	
	**Dominant**	**Non-dominant**	**Dominant**	**Non-dominant**	**Dominant**	**Non-dominant**
**Hand grip strength, kg**						
Mean	25.69	23.75	42.25	39.09	0.000 (–30.24)	0.000 (–30.11)
Median (IQR)	25.4 (22.4∼29)	23.5 (20.8∼26.7)	42 (37∼47)	39 (34.05∼43.7)		
Standard deviation	4.57	4.36	8	7.43		
Maximum	39.8	37.3	70.6	63.1		
Minimum	14.3	11.6	16	19		
*P*-value (*Z*)*	0.000 (–7.04)		0.000 (–8.74)			
**Forearm circumference, cm**						
Mean	22.27	22	25.49	24.92	0.000 (–25.31)	0.000 (–24.23)
Median (IQR)	22 (21∼23.4)	22 (21∼23)	25.5 (24.2∼27)	25 (23.6∼26)		
Standard deviation	1.78	1.76	1.96	1.94		
Maximum	30	30	34	31.9		
Minimum	17	15	20	19		
*P*-value (*Z*)*	0.014 (–2.46)		0.000 (–6.38)			

Definition of abbreviation: IQR, interquartile range.

*Dominant vs Non-dominant. **Men vs Women.

### 3.2. Bivariate correlation analysis

**FIGURE 1:**
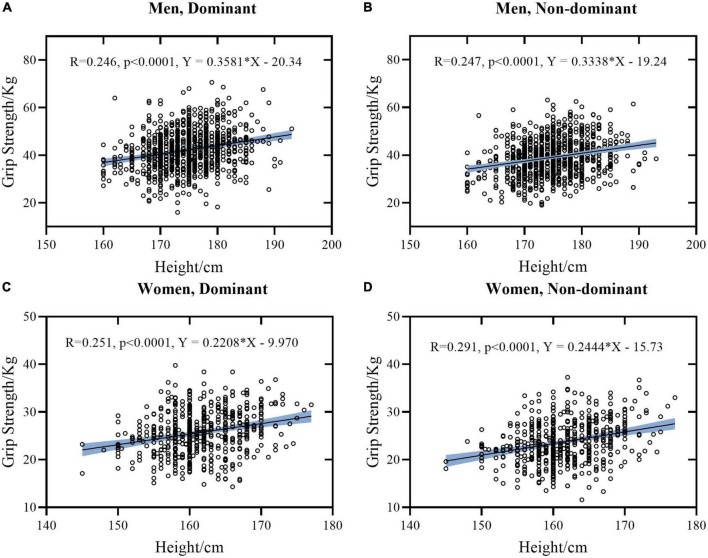
Linear regression models between Height and HGS stratified by sex and hand. **(A)** Data of dominant hand of men. **(B)** Data of non-dominant hand of men. **(C)** Data of dominant hand of women. **(D)** Data of non-dominant hand of women. The light blue area represents the 95% confidence interval.

**FIGURE 2:**
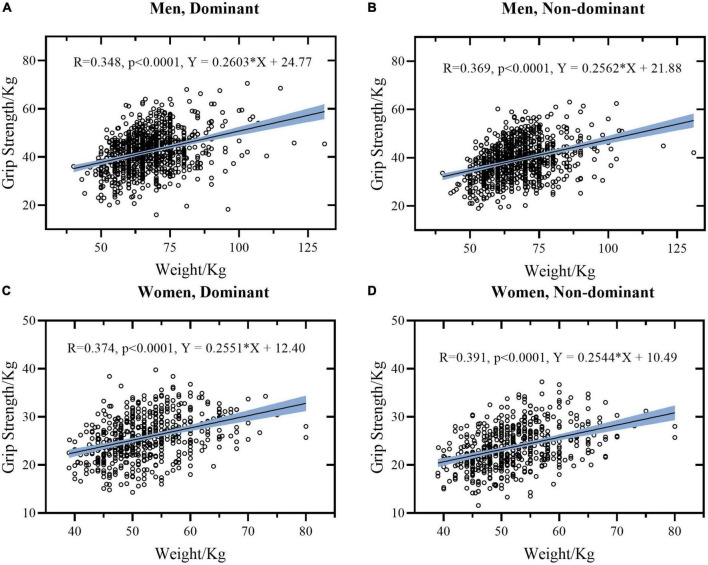
Linear regression models between Weight and HGS stratified by sex and hand. **(A)** Data of dominant hand of men. **(B)** Data of non-dominant hand of men. **(C)** Data of dominant hand of women. **(D)** Data of non-dominant hand of women. The light blue area represents the 95% confidence interval.

**FIGURE 3:**
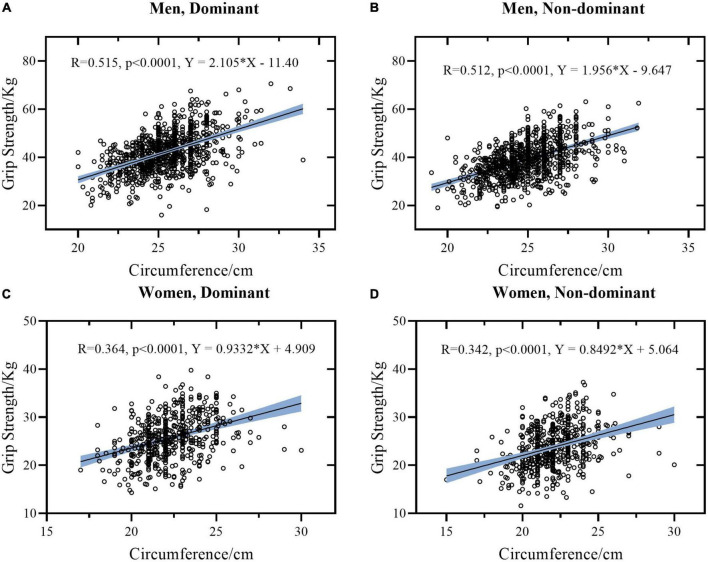
Linear regression models between circumference and grip strength stratified by sex and hand. **(A)** Data of dominant hand of men. **(B)** Data of non-dominant hand of men. **(C)** Data of dominant hand of women. **(D)** Data of non-dominant hand of women. The light blue area represents the 95% confidence interval.

The Pearson or Spearman correlation coefficients were calculated with data of women and men separated (Table 3) and mixed (Table 4) for comparison. Linear regression models between height and HGS, weight and HGS, and FCF and HGS stratified by sex, and hands were presented separately in Figures 1–3. To avoid data duplication in the main text and balance the article length, the remaining graphical representation of correlations and linear regression models were provided in the Supplementary material Appendix B, Supplementary Figures 1–5.

**TABLE 3:** Pearson correlation coefficients between all variables for women (lower left) and men (upper right), separately.

Correlations	Height	Weight	Age	FCF of DH	FCF of NDH	BMI	HGS of DH	HGS of NDH
Height	1	0.504*	-0.004	0.243*	0.262*	0.127*	0.246*	0.247*
Weight	0.474*	1	0.022	0.738*	0.761*	0.919*	0.348*	0.369*
Age	-0.120**	-0.056	1	0.123*	0.125*	0.028	0.080	0.075
FCF of DH	0.176*	0.671*	-0.019	1	0.925*	0.743*	0.515*	0.528*
FCF of NDH	0.147*	0.638*	0.039	0.942*	1	0.760*	0.470*	0.512*
BMI	-0.015	0.779*	0.024	0.593*	0.571*	1	0.291*	0.316*
HGS of DH	0.251*	0.374*	0.029	0.364*	0.348*	0.258*	1	0.806*
HGS of NDH	0.291*	0.391*	0.038	0.350*	0.342*	0.262*	0.829*	1

Definition of abbreviation: FCF, forearm circumference; DH, dominant hand; NDH, non-dominant hand; BMI, body mass index. HGS, hand grip strength.

*Correlation is significant at or under the 0.001 level (2-tailed). **Correlation is significant at or under the 0.001 level (2-tailed).

**TABLE 4:** Pearson and Spearman correlation coefficients between all variables for all participants.

Correlations	Height	Weight	Age	FCF of DH	FCF of NDH	BMI	HGS of DH	HGS of NDH	sex
Height	1								
Weight	0.708*^a^	1							
Age	-0.202*^a^	-0.13*^a^	1						
FCF of DH	0.594*^a^	0.81*^a^	-0.089*^a^	1					
FCF of NDH	0.574*^a^	0.81*^a^	-0.063	0.956*^a^	1				
BMI	0.289*^a^	0.877*^a^	-0.045	0.709*^a^	0.72*^a^	1			
HGS of DH	0.679*^a^	0.633*^a^	-0.13*^a^	0.717*^a^	0.68*^a^	0.41*^a^	1		
HGS of NDH	0.682*^a^	0.644*^a^	-0.13*^a^	0.72*^a^	0.696*^a^	0.422*^a^	0.918*^a^	1	
Sex^#^	-0.767*^b^	-0.661*^b^	0.224*^b^	-0.651*^b^	-0.624*^b^	-0.339*^b^	-0.778*^b^	-0.775*^b^	1

Definition of abbreviation: FCF, forearm circumference; DH, dominant hand; NDH, non-dominant hand; BMI, body mass index; HGS, hand grip strength. *Correlation is significant at or under the 0.001 level (2-tailed). ^*a*^Pearson correlation coefficient. ^*b*^Spearman correlation coefficient. ^#^0 = man and 1 = woman.

Generally, it was found that height, weight, FCF, and HGS were significantly inter-correlated (*p* ≤ 0.001), except for age. Correlations between these measures tended to be similar for women and men, except for correlation coefficients between age and FCF (*r* = –0.019 and 0.039, *p* > 0.001 for women while *r* = 0.123 and 0.125, *p* ≤ 0.001 for men, respectively). Individually, there was a moderate positive correlation between height and weight (*r* = 0.474 in women and *r* = 0.504 in men, respectively). Height was weakly correlated with HGS and FCF in both hands (*r* = 0.147∼0.291 in women and *r* = 0.243∼0.262 in men, respectively). By contrast, weight was strong correlated with FCF (*r* = 0.671 and 0.638 in women and *r* = 0.738 and 0.761 in men, respectively), and was weakly correlated with HGS (*r* = 0.374 and 0.391 in women and *r* = 0.348 and 0.369 in men, respectively).

There is a good correlation between FCF and HGS. It is worth mentioning that when FCF was used to predict HGS, there were differences between women and men. FCF was weakly correlated with HGS (*r* = 0.364 DH and 0.342 for NDH, respectively) in women and was moderately correlated with HGS (*r* = 0.515 for DH and *r* = 0.512 for NDH, respectively) in men. According to Figure 3, when the FCF is 25 cm, the predicted HGS is 28.239 Kg in DH and 26.694 Kg in NDH for women, while the predicted HGS is 41.225 kg in DH and 39.253 kg in NDH for men. This indicates that women have less muscle per unit cross-sectional area of the forearm than men.

There were good correlations between the data of DH and NDH. The FCF of DH was highly correlated with that of NDH (*r* = 0.942 in women and *r* = 0.925 in men, respectively). The HGS of DH was strongly correlated with that of NDH (*r* = 0.829 in women and *r* = 0.806 in men, respectively).

We observed that mixing or without mixing the data of women and men confounded the bivariate correlation analyses (Tables 3, 4). When data were mixed (Table 4), there was a moderate correlation between HGS and height and weight (*r* = 0.633∼0.682), and the degree of correlation was similar. However, when the data of women and men were analyzed separately (Table 3), there was a weak correlation between HGS and height and weight (*r* = 0.246∼0.391). Furthermore, sex was strongly correlated with height (*r* = –0.767) and HGS (*r* = –0.778 for DH and *r* = –0.775 for NDH, respectively) in Table 4. As a comparison, there was a moderate correlation between sex and weight (*r* = –0.661). Moreover, it was found in Tables 3, 4 that age showed no evident correlations with HGS since the effect size of the age span was not big enough.

### 3.3. Partial correlation analysis

Tables 3, 4 revealed that height, weight, and FCF significantly correlated with HGS. Meanwhile, there were significant correlations between height and weight, and between weight and FCF. We performed a partial correlation analysis to explore the actual correlation between HGS, height, weight, and FCF. Analysis results revealed no significant correlation between height and HGS after eliminating the weight effect (rP = 0.090 for DH and 0.130 for NDH in women, and rP = 0.087 for DH and 0.076 for NDH in men, respectively). In addition, the correlation between weight and HGS decreased significantly (from *r* = 0.374 for DH and 0.391 for NDH to rP = 0.188 for DH and 0.238 for NDH in women) or even no significant correlation was observed (*r* = –0.057 for DH and –0.037 for NDH in men) after eliminating the FCF effect. A more extensive overview of these results is presented in Table 5.

**TABLE 5:** Partial correlation coefficients (r_*P*_) between HGS, height, weight, and FCF.

Partial correlation	Control variables	Women				Men			
		**DH**		**NDH**		**DH**		**NDH**	
		**Bivariate**	**Partial**	**Bivariate**	**Partial**	**Bivariate**	**Partial**	**Bivariate**	**Partial**
Height vs HGS	Weight	0.251*	0.090^a^	0.291*	0.130^b^	0.246*	0.087^c^	0.247*	0.076^e^
	FCF		0.204*		0.259*		0.145*		0.136*
Weight vs HGS	Height	0.374*	0.299*	0.391*	0.300*	0.348*	0.267*	0.369*	0.292*
	FCF		0.188*		0.238*		-0.057^d^		-0.037^f^
FCF vs HGS	Weight	0.364*	0.164*	0.342*	0.131^g^	0.515*	0.409*	0.512*	0.409*
	Height		0.335*		0.317*		0.485*		0.498*

Definition of abbreviation: HGS, hand grip strength; FCF, forearm circumference; DH, dominant hand; NDH, non-dominant hand. ^a^*p* = 0.034. ^b^*p* = 0.002. ^c^*p* = 0.007. ^d^*p* = 0.081. ^e^*p* = 0.019. ^f^*p* = 0.248. ^g^*p* = 0.002. *Correlation is significant at or under the 0.001 level (2-tailed).

### 3.4. Multivariate linear regression analysis

A multivariate linear regression analysis was performed to quantitatively compare the influence of height, weight, age, and sex on HGS, adding them as fixed factors (Table 6). The addition and omission of one regression term had to significantly change the *r*^2^ (used for variance explanation) and not cause too high VIF values (used for collinearity diagnosis) of the regression model. The four factors (height, weight, age, and sex) explained 62.5 and 62.8% (*R*^2^) of the variance in the HGS of DH and NDH, respectively.

**TABLE 6:** Multivariate linear regression analyses of HGS.

Hand (Summary)	Parameter	Unstandardized		Standardized	*T*	*P*-value	95% CI		Collinearity statistics	
		*B*	Standard error	Beta			Lower	Upper	Tolerance	VIF
Dominant (*R*: 0.791, Adjusted *R*^2^: 0.625)	(Constant)	-3.083	6.534	-	-0.472	0.637	-15.899	9.733	–	–
	Height (cm)	0.130	0.035	0.102	3.658	<0.001	0.060	0.199	0.317	3.157
	Weight (kg)	0.214	0.020	0.243	10.786	<0.001	0.175	0.253	0.490	2.039
	Age (yr)	0.413	0.148	0.045	2.800	0.005	0.124	0.702	0.945	1.058
	Sex (0/1)^#^	-11.881	0.545	-0.541	-21.811	<0.001	-12.950	-10.813	0.403	2.480
Non-dominant (*R*: 0.793, Adjusted *R*^2^: 0.628)	(Constant)	-4.097	6.041	–	-0.678	0.498	-15.947	7.752	–	–
	Height (cm)	0.122	0.033	0.103	3.711	<0.001	0.057	0.186	0.317	3.157
	Weight (kg)	0.214	0.018	0.261	11.644	<0.001	0.178	0.250	0.490	2.039
	Age (yr)	0.377	0.136	0.045	2.764	0.006	0.109	0.644	0.945	1.058
	Sex (0/1)^#^	-10.751	0.504	-0.527	-21.346	<0.001	-11.739	-9.764	0.403	2.480

Definition of abbreviation: HGS, hand grip strength; CI, confidence interval; VIF, variance inflation factor. ^#^0 = man and 1 = woman.

When comparing the normalized regression coefficients, it was found that sex is the primary factor influencing HGS (Beta = –0.541, *p* < 0.001 in DH and Beta = –0.527, *p* < 0.001 in NDH, respectively), followed by weight (Beta = 0.243, *p* < 0.001 in DH and Beta = 0.261, *p* < 0.001 in NDH, respectively). The regression analyses result in the following predictive equations of HGS:

*Dominant hand* (kg) = –3.083 (–11.881 if woman) + 0.413 × age (yr) + 0.130 × height (cm) + 0.214 × weight (kg).

*Non-dominant hand* (kg) = –4.097 (–10.751 if woman) + 0.377 × age (yr) + 0.122 × height (cm) + 0.214 × weight (kg).

## 4. Discussion

### 4.1. General findings

In both women and men, the correlates of higher HGS include higher height, heavier weight, being men and DH, and larger FCF. This is in line with previous studies (3, 15, 38, 39). We found significant sex and hand differences in HGS and FCF among men and women. Averagely, men were about 65% stronger in HGS and about 13% bigger in FCF than women. Meanwhile, the DH was about 8% stronger and about 2% bigger than NDH in both women and men.

When comparing our HGS with normative data of populations aged about 20 years in other regions and countries, regional and state differences were observed. Notably, the mean HGS of DH of men was similar (42.25 kg vs. 41.5 kg), while for women, it was slightly lower than (25.69 kg vs. 28.4 kg) the mean normative data of their counterparts in Dodds RM’s study (15, 38). Globally, our sample was weaker than Europe (26) and Northern American populations, similar to the population of America, and stronger than the African population. Part of the reference data was obtained from Figure 2 of Dodds RM’s study (15). This is consistent with the findings of Darryl et al. that the HGS values from Europe/North America is the highest, from South Asia, Southeast Asia and Africa is the lowest, and from China, South America, and the Middle East is in the middle (35). Notably, although the study population was all Chinese, the mean HGS of our sample was averagely slightly weaker than (26.61 vs. 25.69 kg in DH of women and 43.99 vs. 42.25 kg in DH of men) that of a sample of 255,157 healthy students in Jiangsu province which located in the east coast of China (30) and was similar with their counterpart of a Taiwan Chinese population (40). These findings emphasize the influence of region and race on variations in HGS and reveal marked heterogeneity in muscle strength of people living in different countries and country-income settings.

### 4.2. Forearm circumference predicts grip strength

According to Tables 3, 4, the correlation coefficient between HGS and FCF was the largest among all variables. This can be explained by the fact that the FCF directly predicts the size of the forearm muscle, and muscle strength is closely related to muscle size (41). Meanwhile, considering that HGS is directly affected by the neural, muscular, and skeletal systems (9), the measurement of HGS is inevitably affected by subjective factors like the communication ability of the researchers and the willingness to cooperate of the participant. Therefore, when a dynamometer is not available, or the participant is unwilling or unable to complete the HGS measurement test, measuring the FCF of the participant could be a more convenient and objective alternative to assess the forearm muscle strength or nutrition status in clinical practice, just as the measuring of calf circumference (5).

### 4.3. Weight is more correlated with hand grip strength than height

According to the correlation and regression analysis results, no significant correlation between height and HGS was observed after eliminating the weight effect (Table 5). This is consistent with previous correlation analyses of adolescents and undergraduate students, like an Indian collegiate population aged 18–25 years (29), 2,239 undergraduate students of a Chinese population aged 18–22 years (30), 94 undergraduate students aged 19–24 years (despite women and men data were mixed for analysis in their study) (12) and 137 men participants of the Korean population age 13–77 years (31). Their correlation coefficients between weight and HGS were greater than between height and HGS. However, partial correlation and regression analysis were not performed to compare the influence of height and weight on HGS in their studies.

In contrast, the correlation analysis results are contradictory in studies that included healthy children and adolescents (22–24, 32, 34), elderly Malaysians (20), hospitalized Portuguese patients (3), healthy Caucasian adults (25–28), healthy Indian collegiate population (33) and healthy Brazilian adults (21). Some studies reported that the correlation coefficient between HGS and height is larger than that between HGS and weight (3, 20, 22, 25–28, 34). While other studies reported that the correlation coefficient is smaller (12, 29–31) or similar (10, 32, 33). It appears that the correlation coefficient between HGS and weight is generally larger than that between HGS and height among adolescents and undergraduate students.

The explanation for the above contradiction can be twofold. First, data of women and men were mixed for analysis in these studies. However, sex is one of the strongest influencing factors on HGS (1, 8). As shown in Tables 1, 2, men are significantly taller than women and significantly stronger than women in HGS. When mixing data of women and men, a positive correlation between height and HGS will inevitably be established through the sex effect. Therefore, mixing the data will inevitably influence the correlation conclusions. The second explanation is the heterogeneity of age and health in their study population. We have stated in the introduction that most of their study population comes from complex and diverse populations, ranging from children to older adults. However, children and adolescents are usually at the stage of physical development, while older adults are usually vulnerable to some common diseases like sarcopenic obesity (42) or sarcopenia (7). Thus, their grip function and physical condition are consequently different.

### 4.4. Strengths and limitations

There are several notable strengths to this study. First, it appears to be the first study to compare the influence of height and weight on HGS in detail by combining correlation, partial correlation, and regression analysis, and the first study to report that weight is more related to HGS than height based on a large-scale sample of healthy undergraduate students. Second, in addition to HGS, this study provided the reference values of FCF, height, and weight of healthy undergraduate students mainly aged 19–21 years. Last, the predictive equations of HGS and graphical linear regression models between HGS, age, sex, height, weight, BMI, and FCF were also provided.

Despite the above strengths, we should also mention some limitations of this study. First, we did not follow a standardized testing position for measuring grip strength as advocated by the American Society of Hand Therapists. This may affect the comparability of HGS measured in this study to other studies because several testing conditions could influence the measured HGS force, including the position. Second, male participants accounted for a large proportion of our study cohort, which may induce sex bias. However, the sample size of women participants was over 550 and could be considered a large sample to some extent. Third, the study cohort was healthy college students mainly aged 19∼21 years. Therefore, generalizability to other populations may be limited, and we should be careful when using our multiple regression model to predict the HGS of other age groups. Last, we observed that weight was more related to HGS than height among healthy undergraduate students, while the precise mechanisms behind the relationship were not fully revealed. Further studies are needed to explore the exact mechanisms underlying the relationship.

## 5. Conclusion

Our cross-sectional study provided the reference values of HGS and FCF of healthy undergraduate students from Southwest China. These reference values have the potential to inform the clinical assessment of nutrition status and muscular function among the 18–23 years old population. There was a good correlation between FCF and HGS, so measuring FCF could be a more convenient and objective alternative to assess the forearm muscle strength or nutritional status when a dynamometer is unavailable in clinical practice. Most importantly, both the bivariate correlation, partial correlation and multivariate linear regression analysis revealed weight was more related to HGS than height among healthy undergraduate students. This implicates that HGS should be normalized by weight not height for eliminating the influence of individual differences.

## Data availability statement

The raw data supporting the conclusions of this article will be made available by the authors, without undue reservation.

## Ethics statement

The studies involving human participants were reviewed and approved by the Ethical Committee of Chongqing University of Posts and Telecommunications. Written informed consent to participate in this study was provided by the participants’ legal guardian/next of kin.

## Author contributions

TX participated in all processes of this study. XL helped to design the study and data analyses. DW helped to data analyses and wrote the article. YZ and QZ helped to design the study, revise the article, and take the decision to submit the article for publication. JY, JJ, and WL helped to collect the data. WL helped to revise the article. JC helped to design the study. All listed authors have made substantial contributions to this study.
