# Supplementary material for: Hand grip strength should be normalized by weight not height for eliminating the influence of individual differences: Findings from a cross-sectional study of 1,511 healthy undergraduates
Source: Front Nutr. 2023 Jan 18;9:1063939. doi: 10.3389/fnut.2022.1063939 (PMC9890066; doi:10.3389/fnut.2022.1063939)
Supplement: Supplementary file 1 [file Data_Sheet_1.DOCX]

**Standardized Procedure & Script for Grip Strength and Forearm Circumference Measurement (English Version -- v1.0)**

1. **Equipment**

- Soft ruler (See Figure S1.1)
- Calibrated CAMRY digital hand dynamometer (See Figure S1.2)

| 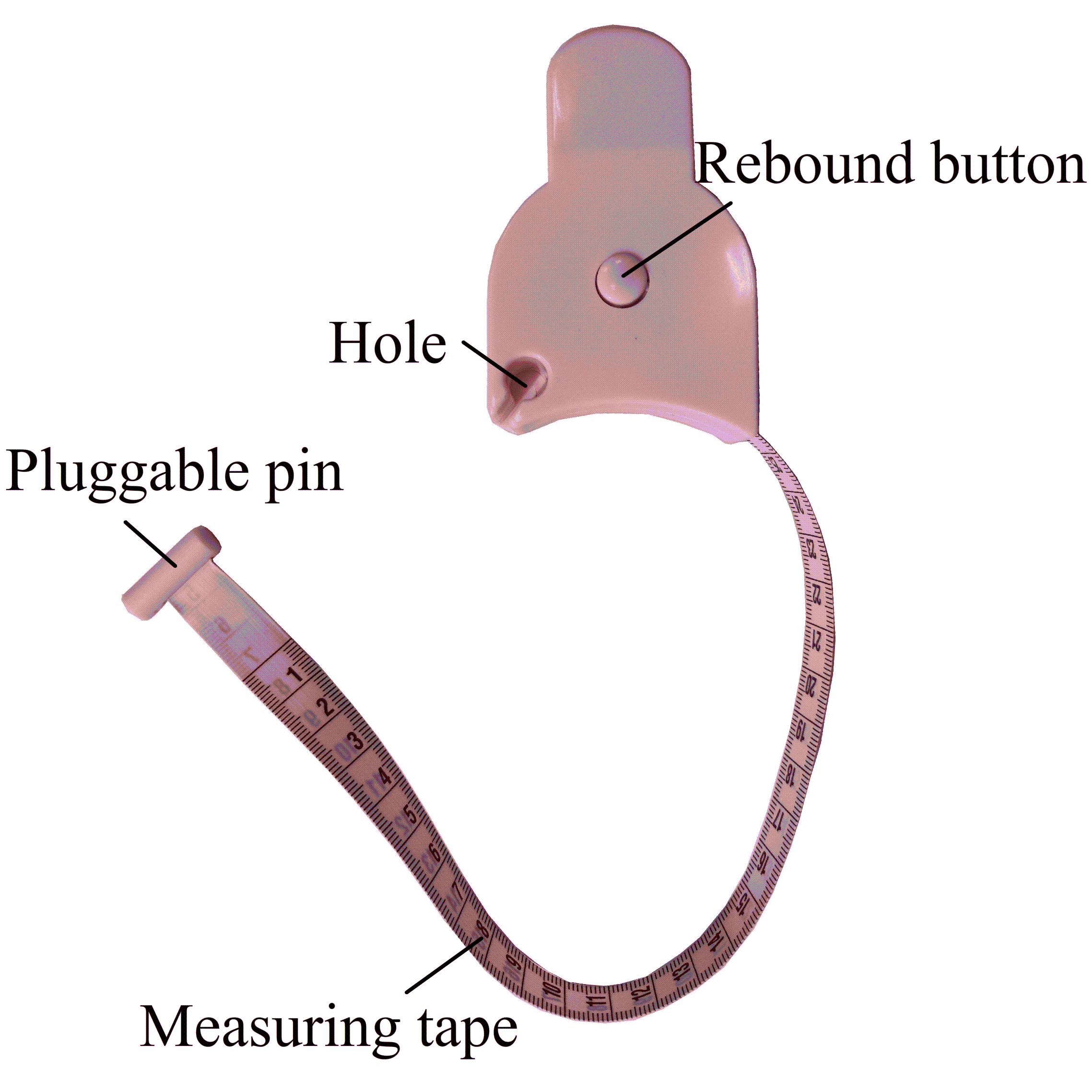 | 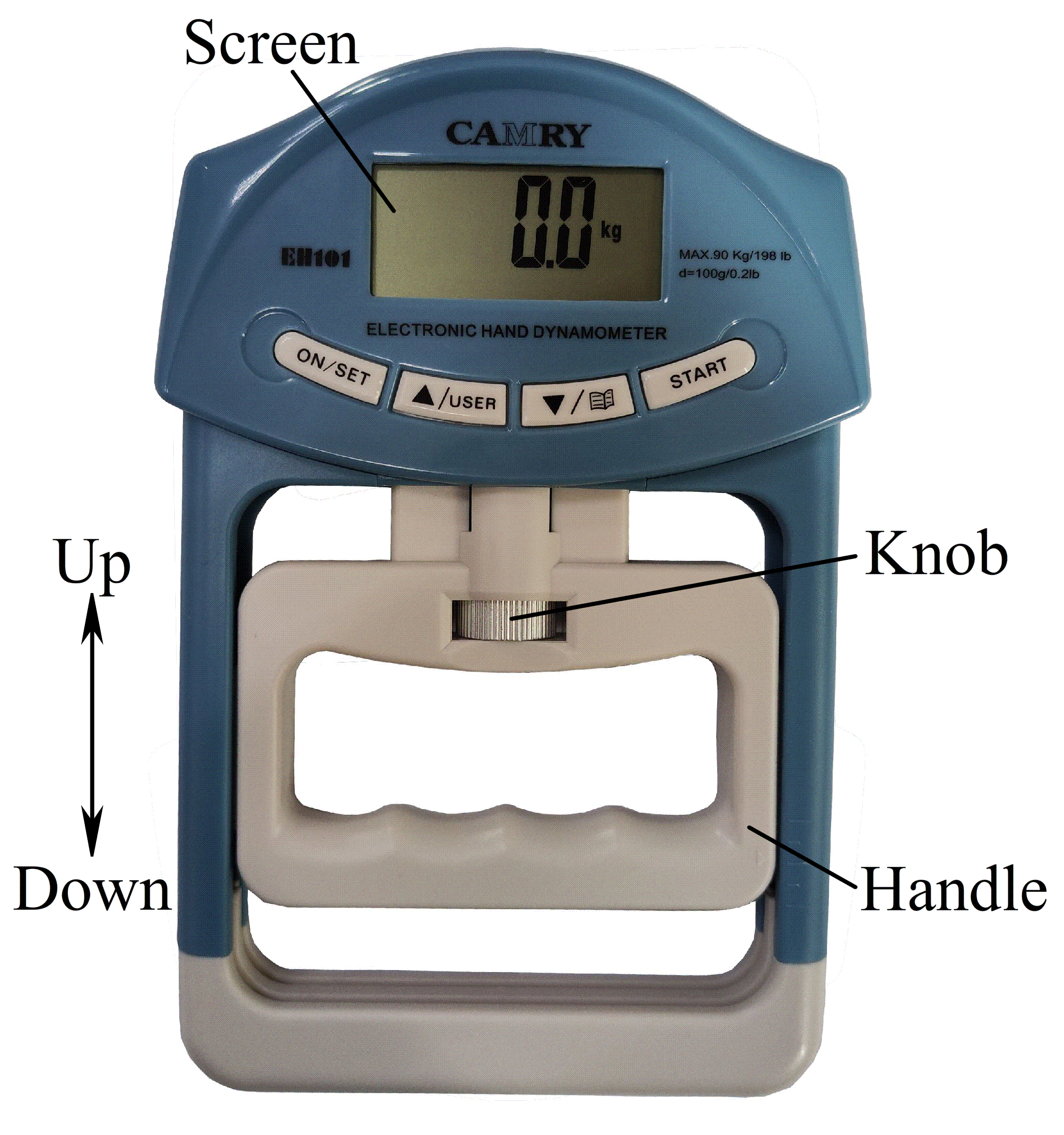 |
| --- | --- |
| Figure S1.1 Soft ruler. | Figure S1.2 Calibrated CAMRY digital hand dynamometer. |

1. **Soft ruler operation**

- Measure both hands and record. First is the right hand and later is the left. Report dominant hand and record.
- Draw out the soft ruler and insert the pluggable pin into the hole to form a measuring ring around the **largest circumference** of the volunteer's forearm. Press the rebound button to attach the soft ruler to the forearm (Figure S1.1).
- Read the scale on the soft ruler with eyes and record. The measuring unit of the soft ruler is in cm, and it is usually estimated to one decimal place (e.g., 25.3cm).

1. **Digital dynameter operation**

- Test both hands and record. First is the right hand and later is the left.
- If the handle does not stay in place (i.e., is too loose), the handle can be moved up and down to the appropriate position by rotating the Knob (Figure S1.2).
- Press the ‘ON/SET’ button to turn on dynamometer.
- Press the ‘START’ button to start measuring.
- Check units on the right of the display read ‘KG’. If it reads ‘LB’, Press ‘ON/SET’ to switch the units to ‘KG’.
- After the measurement, the screen displays the maximum grip during the squeeze automatically.
- When a test is complete, press ‘START’ to reset the display.
- Without use in half a minute, the dynamometer shuts down automatically.

1. **Test procedure and data recording**

**4.1 Forearm Circumference Measurement**

- First is the forearm circumference test of the right hand.
- With the participant in a standing position (See Figure S1.3), say and do the following:

| *“We are going to measure your forearm circumference by using this soft ruler. We need you to keep standing and keep your* *upper limbs relaxed.”*  (Demonstrate the device as it measures forearm circumference) |
| --- |

- Draw out the soft ruler and insert the pluggable pin into the hole to form a ring around the **largest circumference** of the volunteer's forearm. Press the rebound button to attach the soft ruler to the forearm.
- Read the scale on the soft ruler with eyes and record.
- Repeat the above steps to measure the forearm circumference of the left hand.

| 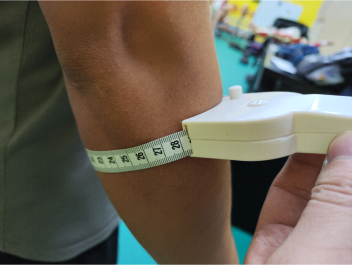 | 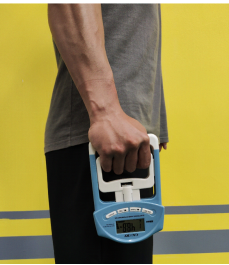 |
| --- | --- |
| Figure S1.3 Example for forearm circumference measurement. | Figure S1.4 Example for grip strength measurement. |

**4.2 Grip Strength Measurement**

- Turn on the dynamometer and participant in a standing position, say and do the following:

| *“We are going to measure your grip strength by having you squeeze this device as hard as you can. The device will not move when you squeeze, but your strength will be recorded automatically by the dynamometer.”*  (Demonstrate the device as it measures force) |
| --- |

- Reset the dynamometer output, pass it to the participant and say:

| *“Now you try just to get a feel for it. For this practice, just squeeze gently. How does that feel?”* |
| --- |

- Testing should be performed with participant in a standing position and with the handle of the dynamometer in the appropriate position. If it is not in the appropriate position, rotate the Knob. For example, if the patient’s hand is very small, rotate the Knob clockwise to move the handle down.
- Reset the output and say:

| *“We are going to measure your strength up to three times to get consistent results. During each test, I want you to gradually squeeze keeping your arm still and without it moving forward or out to the side.”*  (Demonstrate inappropriate motions)  *“Do you have any questions?”* |
| --- |

- First is the maximum grip strength measurement of the right hand.
- Ensure the participant is standing with an upright posture (see Figure S1.3). Keep upper and forearms straight and elbow angle about 180 degrees flexion, and the dynamometer points to the ground (forearm in mid-pronation/supination).
- With the participant correctly positioned as described above, reset the output, pass the participant the dynamometer, and say with consistent tone and volume:

| *“Ready? Squeeze…squeeze…squeeze. Now, stop!”* |
| --- |

- Let the participant squeeze for 3 seconds before saying stop.
- The Trunk should be kept in the same position during the test without leaning forward or backward, and there should be no movement of the arm during the test.
- Record the values displayed on the screen.
- Allow 30 seconds to pass before repeating the test with the participant in the correct positioning and by saying with consistent tone and volume:

| *“Ready? Squeeze…squeeze…squeeze. Now, stop!”* |
| --- |

- Repeat the test for a third time if the first 2 tests do not provide reasonable data (bigger than 10 kg for a healthy woman undergraduate and bigger than 15 kg for a healthy man undergraduate).
- Record the maximum of the grip strength up to 3 trials.
- Repeat the above steps to test the maximum grip strength of the left hand.

**手臂握力和臂围测试标准实验流程（中文版 - 第 1.0版）**

1. **所使用的设备**

- 软尺 (如图 S1.1 所示)
- 测力计(如图 S1.2 所示)

| **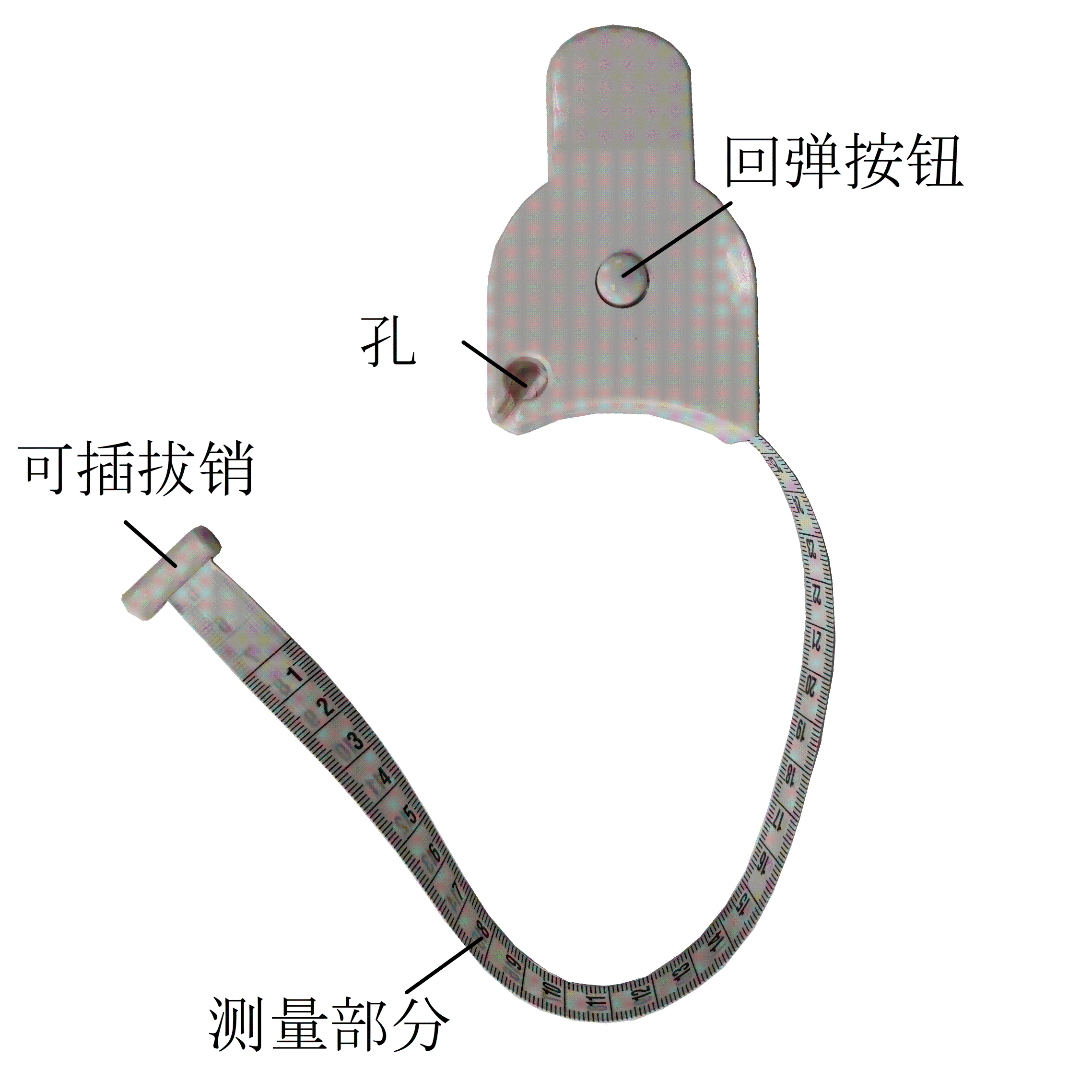** | **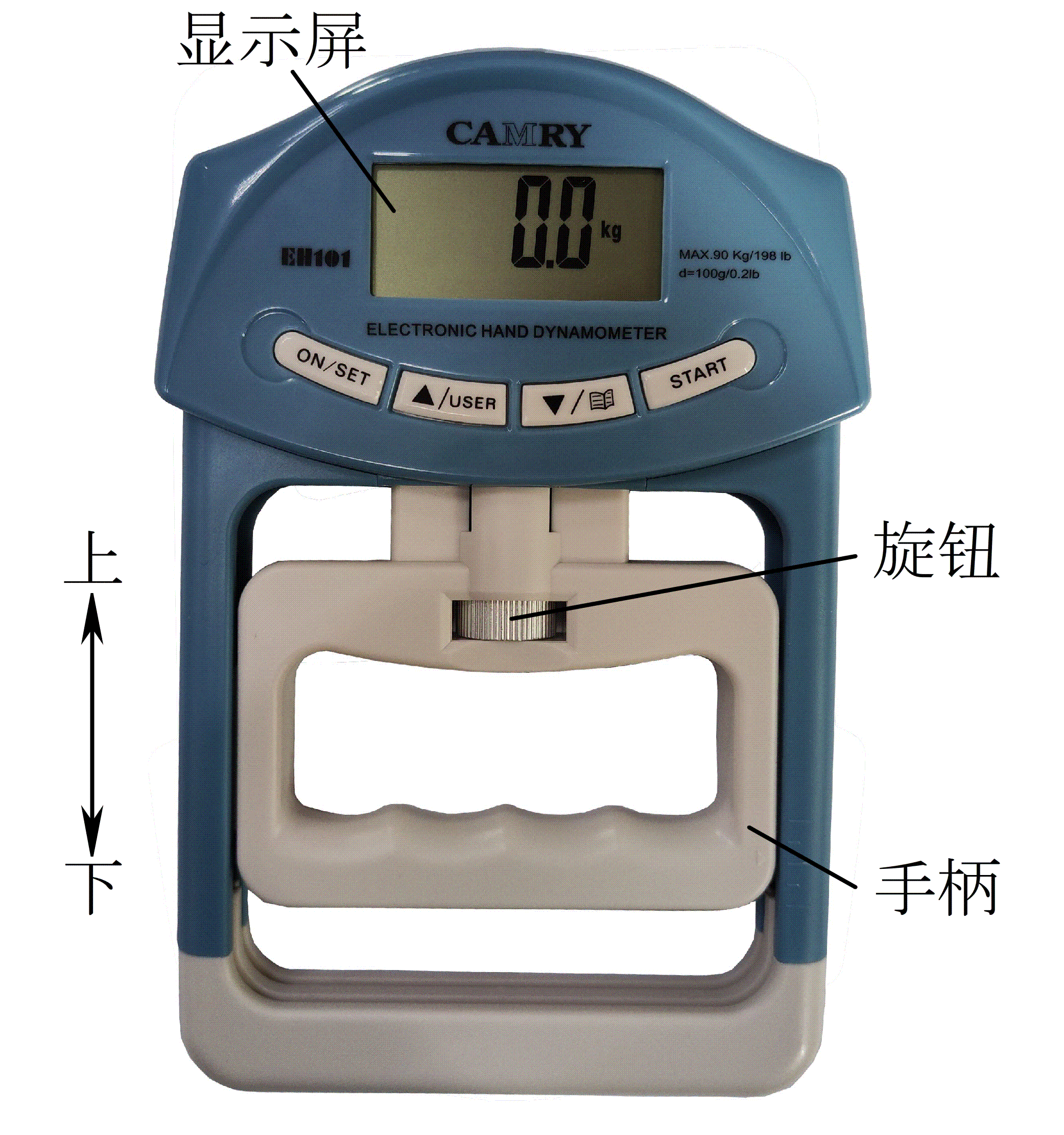** |
| --- | --- |
| **图 S1.1 软尺** | **图 S1.2 测力计** |

1. **软尺操作规范**

- 前后测量志愿者两只手的臂围数据并记录。首先是右手，然后是左手。最后记录志愿者是左撇子还是右撇子；
- 拔出软尺测量部分，将可插拨销插入孔中，在志愿者前臂的**最大臂围处**形成一个测量环。按回弹按钮，将软尺测量部分紧贴在患者前臂上；
- 用眼睛读取软尺上的刻度并记录。注意软尺的测量单位为厘米，通常估计到小数点后一位（如25.3cm）。

1. **测力计操作规范**

- 前后测量志愿者两只手握力并记录。首先是右手，然后是左手；
- 如果手柄未移动到位（如志愿者手过大，造成过于松动），可通过旋转旋钮，使手柄上下移动至适当位置（如图 S1.2所示）；
- 按下“ON/SET”按钮，开启测力计；
- 按“START”按钮开始测量；
- 检查显示屏右侧单位，应当显示“KG”。如果读数为“LB”，按“ON/SET”将单位切换为“KG”；
- 一次测量完成后，屏幕会自动显示实验过程中志愿者的最大握力；
- 测试完成后，按“START”按钮显示屏会清零；
- 半分钟内不使用，测力计会自动关闭电源。

1. **实验流程及数据记录**

**4.1 前臂臂围测试：**

- **首先开始右手的实验**；
- 请志愿者保持站立姿势（如图S1.3所示），按如下流程向志愿者解释并完成操作：

| 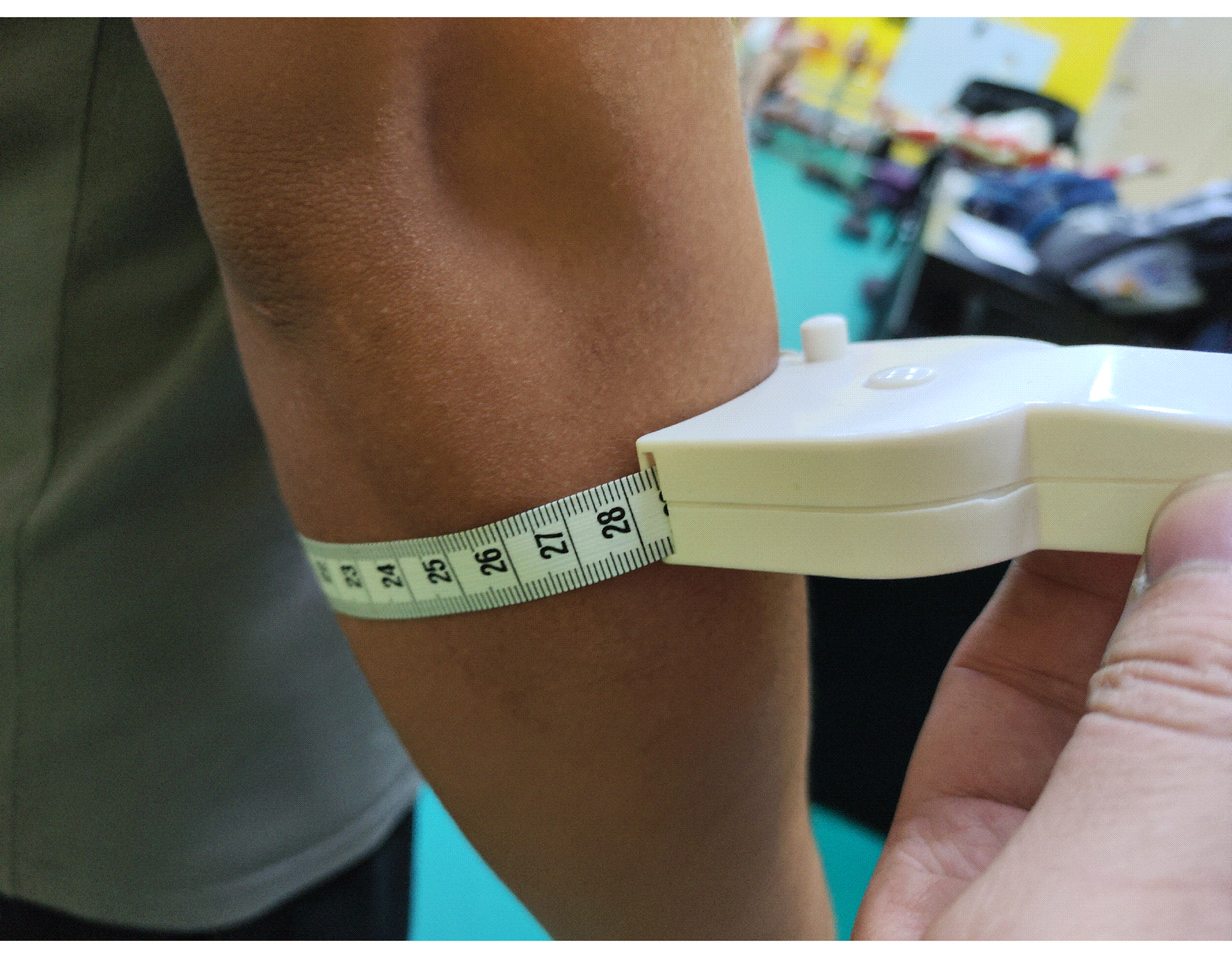 | 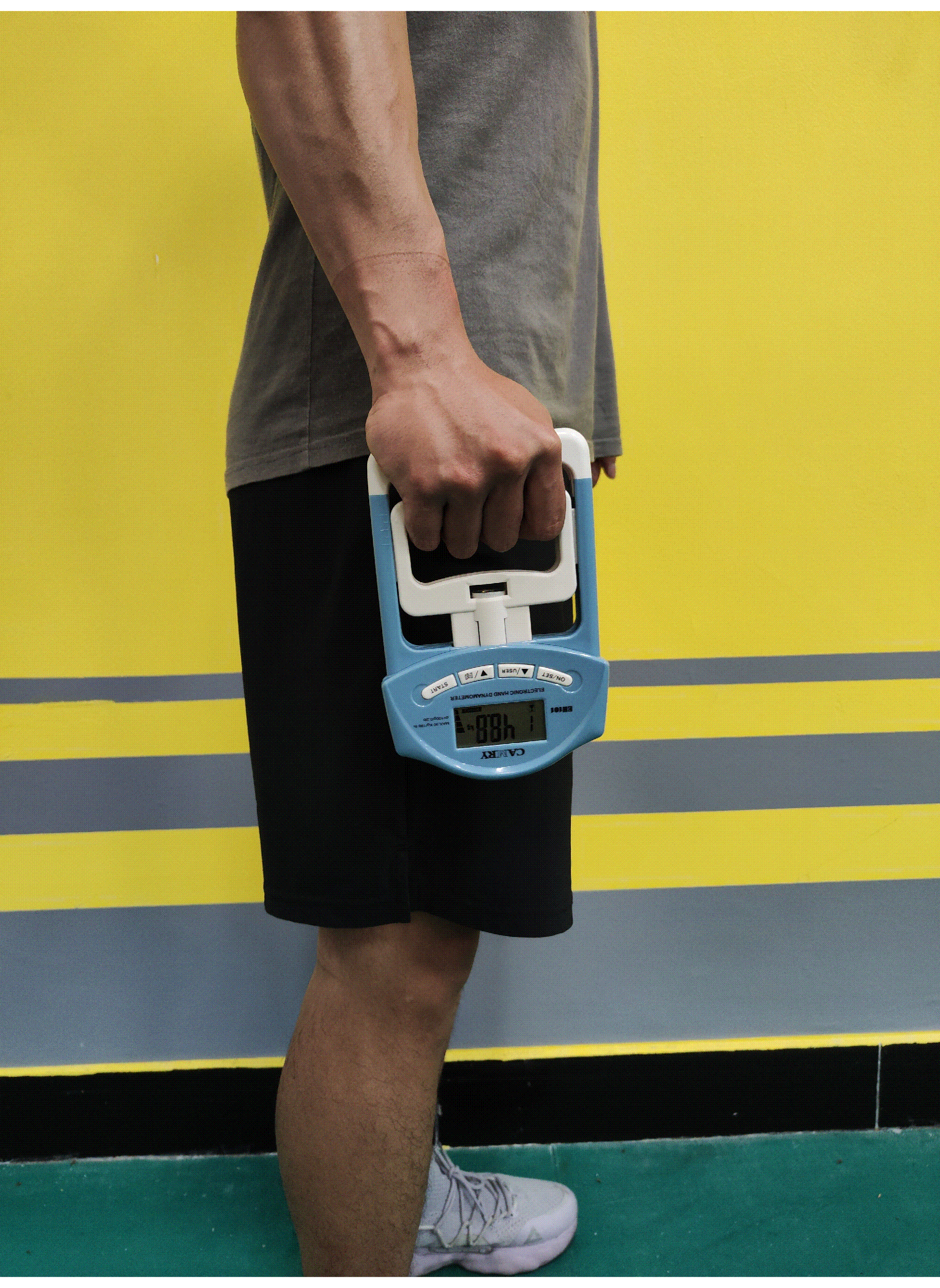 |
| --- | --- |
| **图S1.3 臂围测试示例** | **图S1.4 握力测试示例** |

| *“我们需要使用软尺测量您的前臂周长。请配合保持站立姿势，并保持您的上肢处于放松状态。”*  (向志愿者演示测量前臂周长的设备) |
| --- |

- 抽出软尺测量部分，将可插拨销插入孔中，在志愿者前臂的最大周长处形成一个封闭的圆环。按回弹按钮，将软尺测量部分紧贴在志愿者前臂上；
- 读取软尺测量部分的刻度并记录；
- **重复上述实验步骤，完成左手臂围的测试。**

**4.2 手臂最大握力测试**

- 按测力计上的‘ON/SET’按钮，打开测力计，让志愿者保持站立姿势，按如下流程向志愿者解释并完成操作：

| *“我们需要您按如图S1.4所示那样，尽您最大可能地握测力计的手柄部位，以此来测量您手臂最大握力。测量过程中不需要您参与数据的记录，测力计会自动地记录您握的过程中最大握力值。”*  （向志愿者演示测力计） |
| --- |

- 将测力计设置好以后，将其传递给志愿者，并与志愿者沟通：

| *“现在请您试着熟悉这个设备。在熟悉的过程中，只需要您轻轻握测力计。请问您感觉怎么样？”* |
| --- |

- 测试过程中，志愿者应当一直处于站立姿势且握力计的手臂处在合适的位置。如果志愿者的手比较小，应当顺时针旋转旋钮以向下移动手柄至合适的位置。
- 重新还原测力计并向志愿者沟通到：

| *“为了获得准确的结果，最多需要您完成三次手臂握力的测量。在每次测试中，希望您按照要求逐渐握紧测力计的手柄至最大握力值。过程中请保持手臂静止，不要向前或向外移动。”*  （向志愿者展示不正确的示范）  *“请问您还有其他问题吗？如果没有，将正式开始实验。”* |
| --- |

- **首先开始右手的实验**；
- 确保志愿者保持直立站立的姿势（如图S1.3所示）。确保志愿者上臂和前臂处于伸直的状态，肘关节角度约180度弯曲，测功机指向地面（前臂处于旋前/旋后中间的位置）。
- 确保志愿者在如上所述正确姿势的状态下，重置测力计，将测力计递给志愿者，并以平稳一致的音调和音量引导志愿者：

| *“准备好了吗？请开始用力握手柄，保持……保持……保持。好，可以了！”* |
| --- |

- 确保志愿者握的持续时间超过3秒以后才停止实验；
- 试验过程中，确保志愿者身体保持在同一位置，不向前或向后倾斜，试验过程中不应有手臂的运动；
- 在实验记录表上记录测力计屏幕上显示的最大握力值；
- 确保志愿者在休息30秒以后才开始下一次测试，并同样以平稳一致的音调和音量引导志愿者：

| *“准备好了吗？请开始用力握手柄，保持……保持……保持。好，可以了！”* |
| --- |

- 如果前2次试验没有提供合理范围内的数据，则需要重复试验第3次实验；
- 在试验记录表上记录3次试验中握力的最大值；
- 重复上述实验步骤，完成左手最大握力的测试。
