# Supplementary material for: Hand grip strength should be normalized by weight not height for eliminating the influence of individual differences: Findings from a cross-sectional study of 1,511 healthy undergraduates
Source: Front Nutr. 2023 Jan 18;9:1063939. doi: 10.3389/fnut.2022.1063939 (PMC9890066; doi:10.3389/fnut.2022.1063939)
Supplement: Supplementary file 2 [file Data_Sheet_2.docx]

**Appendix B:**


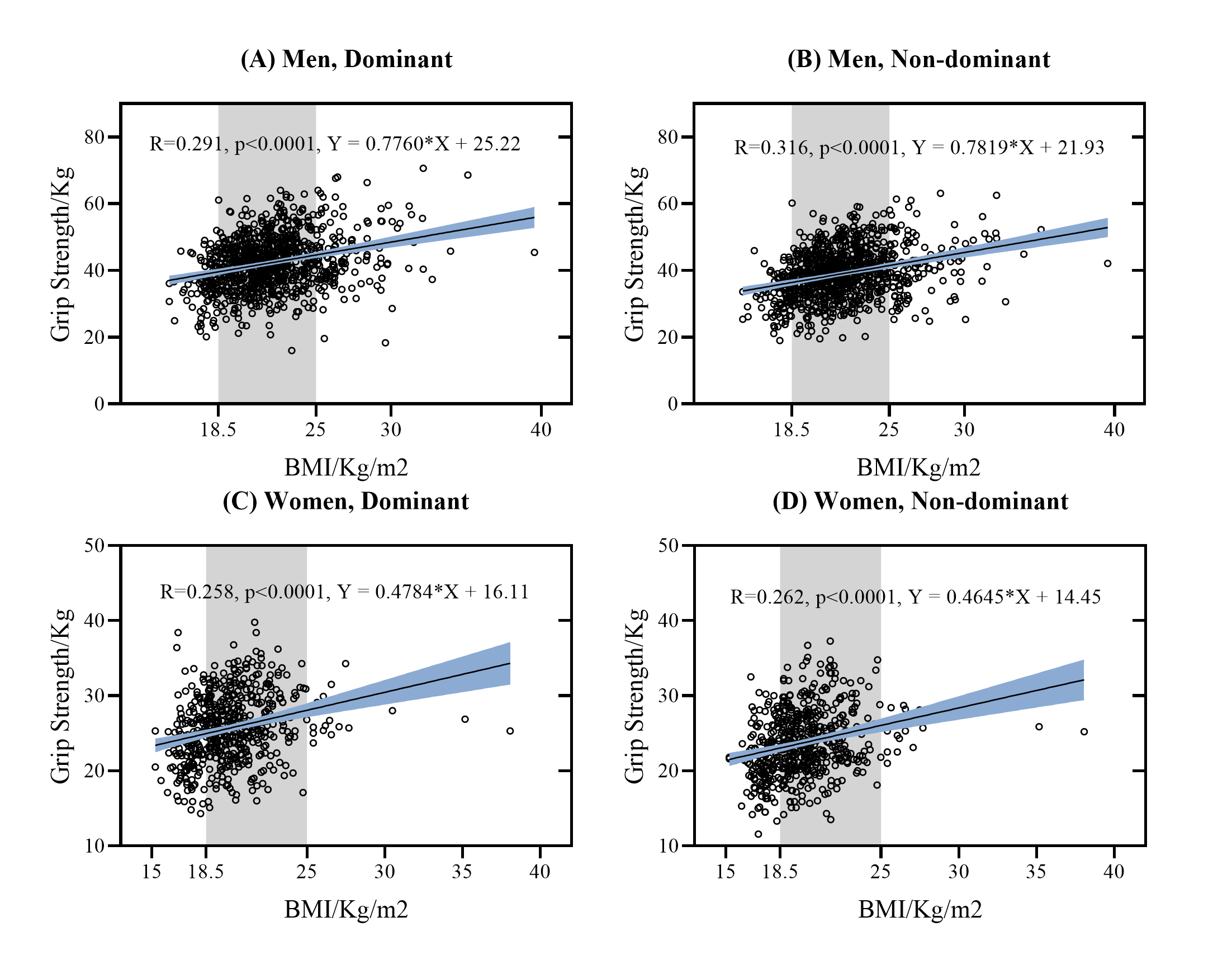


Figure S1 Linear regression models between BMI and HGS stratified by gender and hand. Grey shading shows 18.5<BMI<25 and light blue area represents the 95% confidence interval. Definition of abbreviation: BMI, body mass index. HGS, hand grip strength.


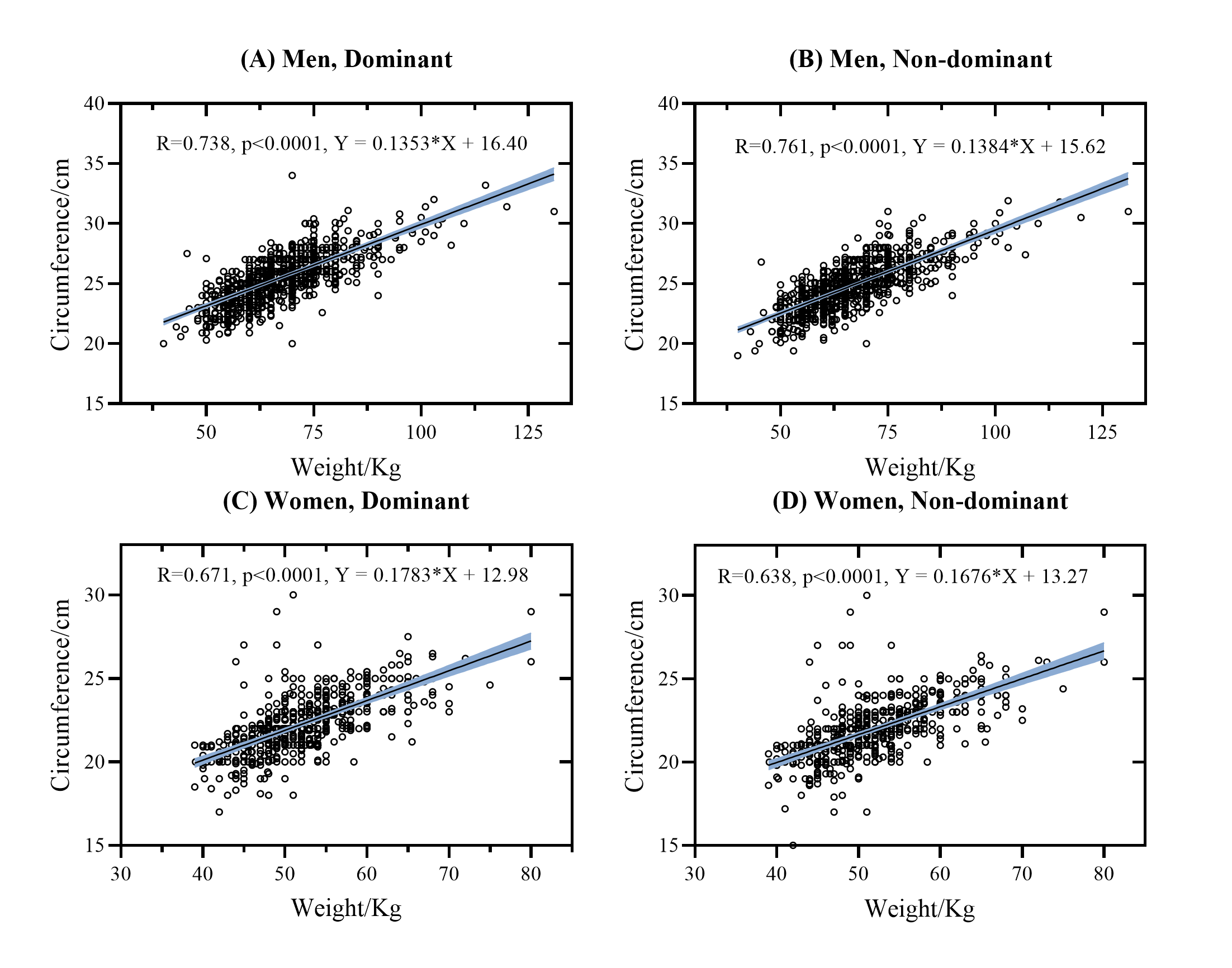


Figure S2 Linear regression models between weight and FCF stratified by gender and hand. Light blue area represents the 95% confidence interval. Definition of abbreviation: FCF, forearm circumference.


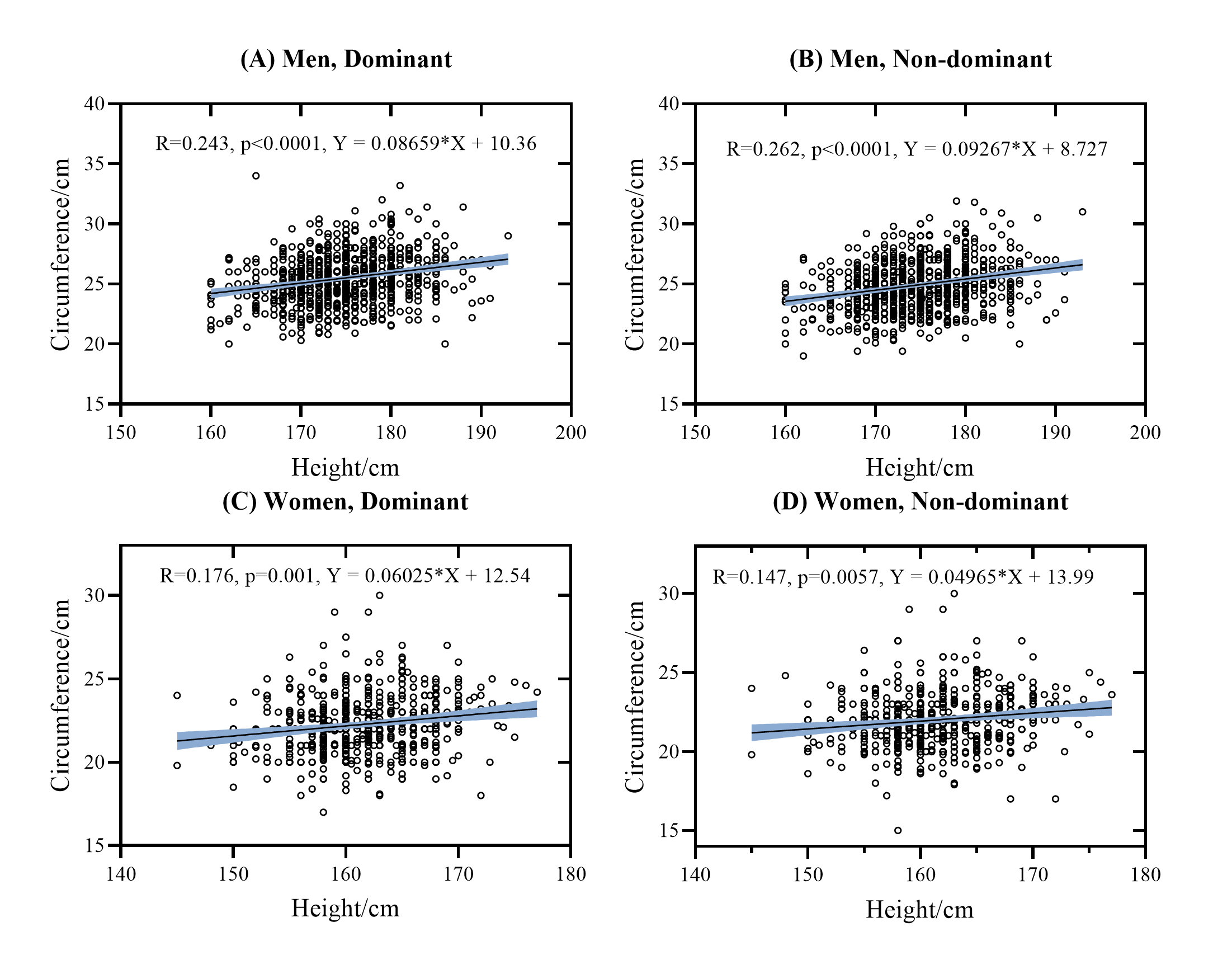


Figure S3 Linear regression models between height and FCF stratified by gender and hand. Light blue area represents the 95% confidence interval. Definition of abbreviation: FCF, forearm circumference.


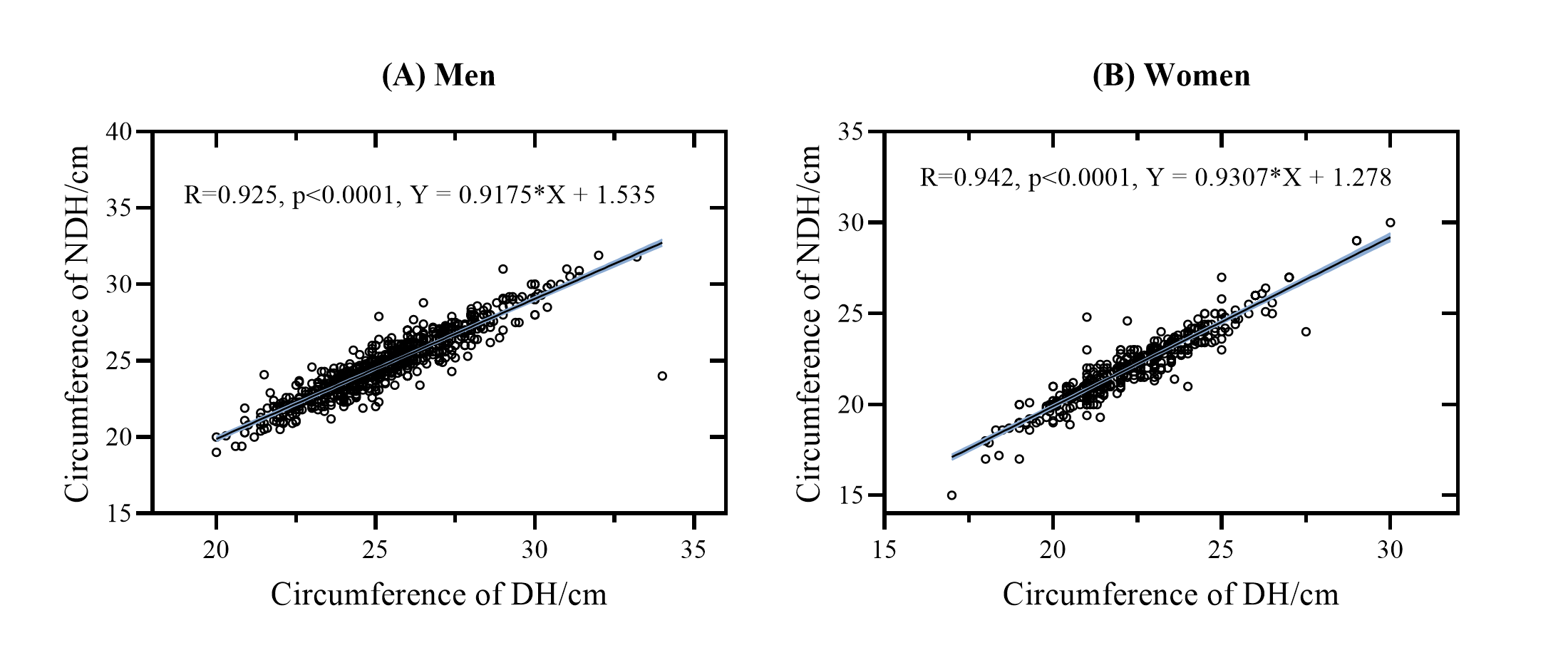


Figure S4 Linear regression models between FCF of DH and NDH stratified by gender. Light blue area represents the 95% confidence interval. Definition of abbreviation: FCF, forearm circumference. DH, Dominant Hand. NDH, Non-Dominant Hand.


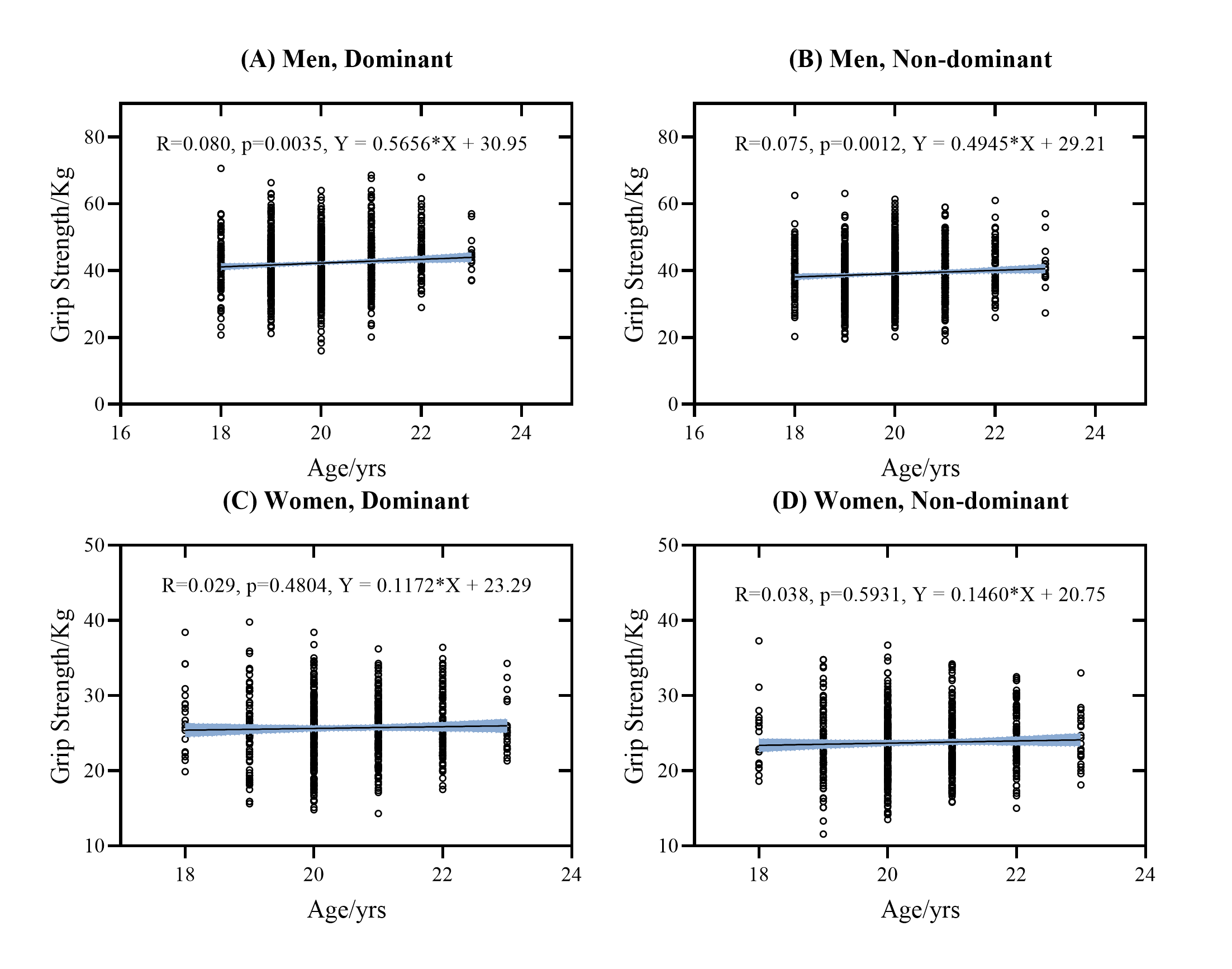


Figure S5 Linear regression models between Age and HGS. Light blue area represents the 95% confidence interval. Definition of abbreviation: HGS, hand grip strength.
